# Targeting visual-sensory and cognitive impairments following lateral ankle sprains: a practical framework for functional assessment across the return-to-sport continuum. Part 2: from theory to practice: recommendations for optimizing return to sport after lateral ankle sprains using cognitive and visual-sensory assessments

**DOI:** 10.3389/fspor.2025.1702858

**Published:** 2025-11-17

**Authors:** Brice Picot, Alexandre Maricot, François Fourchet, Alli Gokeler, Bruno Tassignon, Ronny Lopes, Alexandre Hardy

**Affiliations:** 1 Inter-University Laboratory of Human Movement Biology (LIBM), Savoie Mont-Blanc University, Chambéry, France; 2 French Society of Sports Physical Therapy (SFMKS Lab), Pierrefitte-sur-Seine, France; 3 Human Physiology and Sports Physiotherapy Research Group, Faculty of Physical Education and Physiotherapy, Vrije Universiteit Brussel, Brussels, Belgium; 4 Department of Physiotherapy, La Tour Hospital, Meyrin, Switzerland; 5 Exercise Science and Neuroscience, Department of Exercise & Health, Paderborn University, Paderborn, Germany; 6 Faculty of Health, Amsterdam University of Applied Science, Amsterdam, Netherlands; 7 REVAL Rehabilitation Research Center, Faculty of Rehabilitation Sciences, Hasselt University, Diepenbeek, Belgium; 8 Centre Orthopédique Santy, Hôpital Privé Jean Mermoz, Ramsay Santé, Lyon, France; 9 Clinique Du Sport, Paris, France

**Keywords:** lateral ankle sprain, chronic instability, cognition, sensory reweighting, return to sport, Ankle-GO score

## Abstract

Lateral ankle sprain (LAS) is the most common traumatic injury in sports, characterized by a high recurrence rate, with chronic ankle instability (CAI) developing in ∼40% of cases. Both altered sensory reweighting and cognitive impairments have been identified as potential contributors to the elevated risk of (re)injury. The first part of this work aimed to clarify cognitive constructs relevant to post-injury rehabilitation, alongside the concept of sensory reweighting that may be observed in patients following LAS. It also introduced the Ankle-GO™, the first validated score providing clinicians with an objective criterion to support return-to-sport (RTS) decision-making. However, this promising tool does not account for visual and cognitive constraints encountered during functional tasks. Therefore, the second part of this work aims to translate emerging theories and growing evidence into practical applications, illustrating concrete examples of RTS assessments in patients with LAS and CAI. This perspective's article proposes a “β(rain)” extension of the Ankle-GO™ integrating dual-tasks paradigms and visual constraints to better approximate sport-specific conditions. Each functional test (Single leg stance, modified Star Excursion Balance Test, Side Hop Test and Figure-of-8 test) is paired with either a dual-tasks targeting key cognitive domains or a visual constraint. An adapted scoring method is outlined, together with a guide for interpreting results during the late rehabilitation phase, tailored to patients' specific deficits.

## 1 Introduction

To date, the Ankle-GO™ score represents the only validated tool after lateral ankle sprains (LAS). It allows to distinguish patients who will successfully return to their pre-injury level of play (1), those who will suffer from reinjury (2), and those who will achieve full recovery following LAS (3).

Although its construction followed the recommendations of the International Ankle Consortium for return to sport (RTS) evaluation (4), this cluster of functional test and self-reported questionnaires does not capture the full spectrum of central deficits observed in patients with chronic ankle instability (CAI) and LAS (5). In particular, cognitive and sensory alterations have been consistently identified in these populations and should be explicitly addressed during patient evaluation (6–8). Accordingly, dual-task situations incorporating cognitive demands and visual constraints should be implemented throughout rehabilitation to more effectively assess patient's abilities. In the first part of this work (51), we outlined the theoretical background of central alterations in CAI, described the construction and limitations of the Ankle-GO™ score with respect to these deficits, and discussed the key concepts for evaluating neurocognitive impairments. In this second part, we propose a framework for translating these theoretical concepts into practical clinical applications.

## 2 From theory to practice…

### 2.1 What are the key concepts?

To optimize rehabilitation and enhance the effectiveness of goal-oriented assessments, clinicians may draw on the Functional Task Environment (FTE) framework proposed by Gokeler et al. (9). The FTE emphasizes replicating real-world sport conditions during both evaluation and rehabilitation by considering the dynamic interaction between the task, the environment, and the individual. This approach can be applied from the early stages of recovery through the entire RTS continuum, depending on the patient's capacities and is guided by four key principles:

1. Progress from simple to complex: Begin with controlled movements and gradually introduce unpredictability and sport-specific challenges.
2. Integrate cognitive demands: Incorporate cognitive loads ranging from lower-order to higher-order processes in order to simulate competitive game pressures.
3. Tailor to the sport and athlete: Customize drills to replicate the specific movement patterns, timing demands, and psychological stressors of the athlete's sport.
4. Contextualize return-to-sport decisions: Ensure that athletes can perform under realistic conditions—not only physically, but also cognitively and emotionally.

### 2.2 What should be assessed and how?

To ensure accurate and clinically relevant assessment of cognitive and visual-sensory impairments, several key elements should be considered. Functional tests should first be performed under single-task conditions (“motor” only) and subsequently under dual-task conditions (“motor+cognitive”). Clinicians should then report the results of these assessments using standardized outcomes measures (10). In addition, it is essential to evaluate cognitive performance in quiet, seated condition to establish the patient's baseline abilities.

#### 2.2.1 Motor outcomes

The following outcomes should be reported: Total task completion time, movement quality (e.g., kinematic or kinetic analysis), and static and dynamic balance performance, including postural errors and reached distance on the Star Excursion Balance Test.

#### 2.2.2 Cognitive outcomes

Cognitive domains critical for sport performance include attention, working memory, and inhibitory control. Accordingly, assessment should incorporate the following components:

- Cue-based response tasks: athletes respond to visual or auditory stimuli during movement (e.g., change direction only when a specific cue appears).
- Working memory tasks: modified N-back tasks integrated into agility drills or balance challenges.
- Inhibitory control: Go/No-Go or Stroop-like paradigms integrated into functional tasks.

Key performance metrics include accuracy, response time, and error rate, assessed under both single- and dual-task conditions.

#### 2.2.3 Identify and understand prioritization?

Task prioritization should be a specific focus during assessments, as it reflects how individuals allocate attentional resources across concurrent tasks. To capture this accurately, assessments should incorporate tasks requiring integrated cognitive-motor coordination, which more closely approximate real-world demands. For instance, tasks that involve responding to unpredictable stimuli during movement (e.g., target-directed stepping or decision-making while running) provide more meaningful insights than dual-tasks combining unrelated domains, such as backwards counting during static balance, which may promote artificial rather than natural prioritization. Accordingly, the use of multiple dual-tasks paradigms is recommended. Evaluating how athletes prioritize motor vs. cognitive tasks under these conditions offers valuable insight into neuromuscular control and cognitive resilience following injury:

- Motor/balance First: Athletes may overcompensate for joint instability by prioritizing motor control, potentially at the expense of situational awareness.
- Cognition First: In fast-paced contexts, some athletes may prioritize decision-making, which can compromise joint protection or biomechanical control.
- Flexible Prioritization: Optimal recovery is characterized by the ability to switch flexibly between tasks based on contextual demands, a key marker of RTS readiness.

Grounded in this theorical framework, the present perspective article aimed to provide clinicians with practical strategies for evaluating patients along the RTS continuum following LAS.

### 2.3 Concrete proposition: “β(rain)” extension of the Ankle-Go™ score

**Figure 1:**
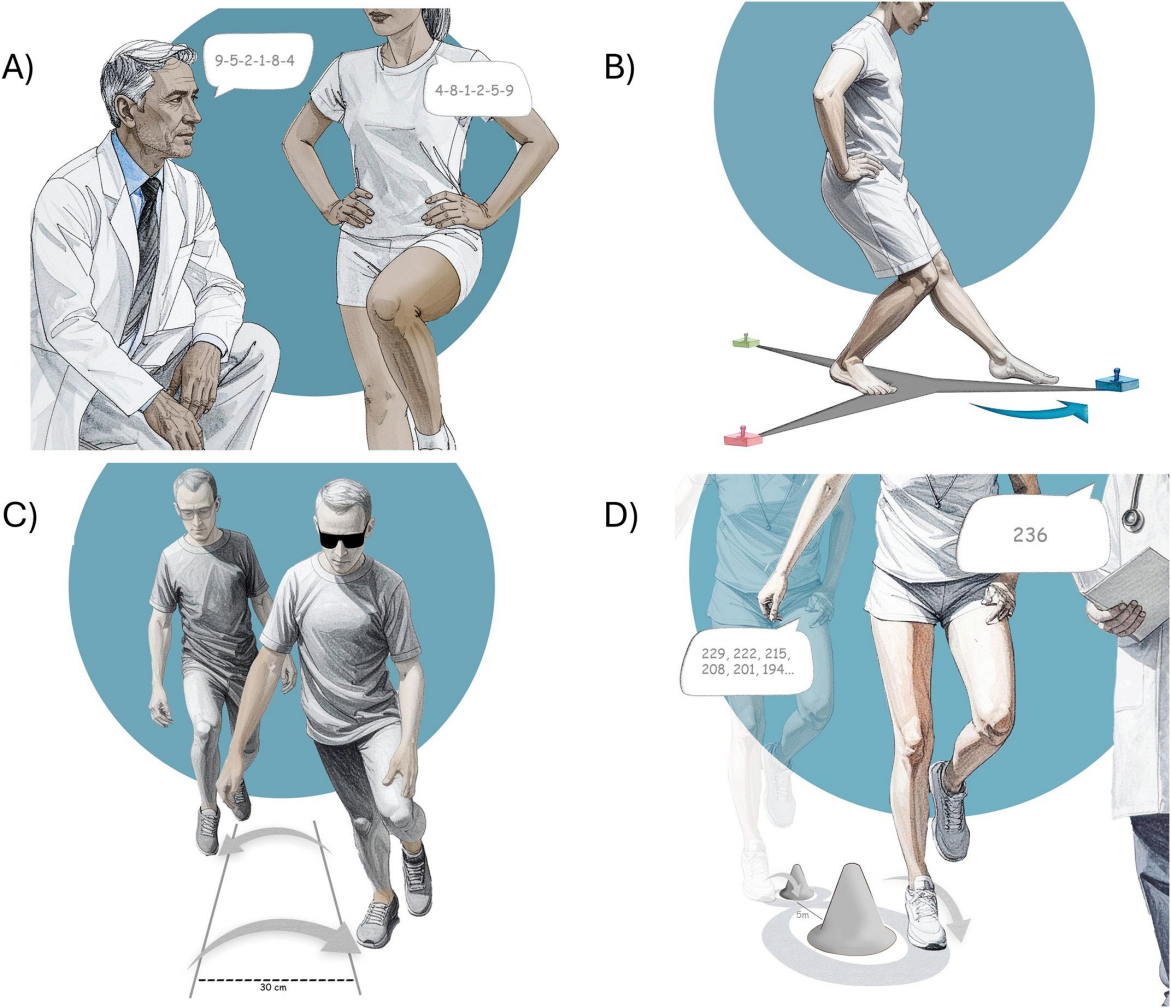
The β(rain) extension of the Ankle-GO™ score. **(A)** Single leg stance combined with digt/word span, **(B)** reactive balance test, **(C)** side hop test under visual constrain, **(D)** Figure-of-8 test with substraction.

We selected four functional tests widely used in RTS decision-making and included in the Ankle-GO™ score - Single Leg Stance, modified Star Excursion Balance Test, Side Hop Test and Figure-of-8 Test (11, 12) - and sought to increase their complexity by incorporating cognitive and visual constraints to enhance external validity (Figure 1). The proposed “β(rain)” extension introduces dual-task paradigms designed to address central impairments commonly observed following LAS but not accounted for in the “classical” version of the Ankle-GO™ score (51). To support clinicians in evaluating and interpreting patient performance, a dedicated scoring system is outlined in Table 1. Cut-off scores were selected based on previous existing literature (13, 14) and preliminary data.

**Table 1:** Example of a proposed β(rain) extension of the Ankle-GO™ scoring system.

Items		Raw scores	Weight	Maximum score
FAAM	Activities of daily living	<90%	0	**2**
		90%–95%	1	
		>95%	2	
	Sport	<80%	0	**2**
		80%–95%	1	
		>95%	2	
ALR-RSI		<55%	0	**3**
		55%–63%	1	
		63%–76%	2	
		>76%	3	
SLS + digit/word span		>3 errors	0	**3**
		1–3 errors	1	
		0 error	2	
		Incorrect response	−1	
		No feeling of instability	+1	
Reactive balance test		VMRT >900 ms	0	**7**
		VMRT 800–900 ms	1	
		VMRT <800 ms	2	
		Accuracy <80%	0	
		Accuracy 80%–85%	2	
		Accuracy >85%	4	
		No feeling of instability	1	
SHT + strobe glasses		>13 s	0	**5**
		10–13 s	2	
		<10 s	4	
		No feeling of instability	+1	
F8T + backward counting		>18 s	0	**3**
		13–18 s	1	
		<13 s	2	
		<4 correct answers	−1	
		No feeling of instability	+1	
β(rain) Ankle-GO™ score				**25**

FAAM, foot and ankle ability measure; ALR-RSI, ankle ligament reconstruction-return to sport after injury; SLS, single leg stance; VMRT, visuo motor reaction time; SHT, side hop test; F8T, figure-of-eight test.

The values in bold indicate the maximum scores that can be achieved for each item and for the total score.

#### 2.3.1 Single leg stance (SLS) + digit/word span

A backward digit or word span task, consisting of random sequences of 6 numbers or words, (e.g., *4-8-1-2-5-9* or *river-cloud-pencil-journey-silent-mirror,* see additional examples in the Supplementary Material 1–2), is presented to the patient during the first 10s of the SLS (14, 15). The subject is then required to repeat the sequence in exact reverse order (i.e., *9-5-2-1-8-4* or *mirror-silent-journey-pencil-cloud-river* in the above examples) during the subsequent 10 s (Figure 1). Participants are instructed only to complete the cognitive task while maintaining balance, as providing more specific instructions has been shown to bias dual-task balance performance (16). This task has been shown to be highly reliable (14). Similarly to the standard SLS, the evaluator records the number of postural errors (1) and notes whether the participant responds correctly. If not, one point is deducted:

#### 2.3.2 Reactive balance test (RBT)

The RBT is a dynamic balance assessment that inherently integrates cognitive-motor interactions (13, 17, 18). Unlike conventional dual-task paradigms in which cognitive and motor tasks are performed simultaneously but independently, the RBT incorporates a cognitive decision-making process that directly influences the motor response, thereby providing a more ecologically valid measure of functional performance.

In the RBT, three LED lights are positioned along each axis of the Y-Balance Test (YBT) or mSEBT — anterior, posteromedial, and posterolateral, with an additional lead LED placed in front of the setup. Each LED displays one of three colors (blue, green, or red), with each color corresponding to a specific axis. The participant is instructed to respond as quickly and accurately as possible to the color cue by reaching with the free foot over the LED located along the indicated axis (17). The LED distances are individualized according to the participant's YBT/mSEBT performance (i.e., 80% of the maximal reach distance for each axis). The test consists of 36 stimuli delivered over approximately 90 s, with 12 stimuli randomly assigned to each axis. Each stimulus must be extinguished by the free foot within two seconds, after which the next stimulus is triggered; faster responses result in a shorter interval between stimuli.

Performance on the RBT is evaluated using 2 metrics (Table 2): visuomotor response time (VMRT, in seconds) and accuracy (expressed as a percentage). VMRT reflects the average time required to extinguish the LEDs across 36 stimuli). Accuracy reflects the number of correct responses, with errors including missed stimuli (failure to extinguish a light within the time limit), incorrect responses (reaching toward the wrong axis), multiple attempts to extinguish the same light, and balance errors (Table 2).

**Table 2:** Reactive balance test outcome measures [from (13)].

Visuomotor response time (in ms)	Averaged total visuomotor response time
Accuracy (in %)	{[Total number of stimuli – (missed stimuli + multiple attempts needed + decision errors + balance errors)]/Total number of stimuli} × 100
Missed stimulus	Failed to extinguish LED-light in less than 2 s
Multiple attempts	Reaching from standardized position, but failed to extinguish the LED-light from the first time; second or third attempt needed
Decision error	Initiating movement in wrong direction
Balance errors	- The participant did not start from the standardized position at stimulus onset
	- The participant is trying to find balance during the reach
	- The participant needs to put a hand or foot on the floor
	- The participant steps off the YBT Test kit or SEBT grid
	- The participant is not able to keep the hands on the hips
	- The participant lifts the forefoot or heel off the testing surface

YBT, Y-balance test; mSEBT, modified star excursion balance test.

It should be noted that the RBT was originally validated using the Fitlight® system, but other reactive training tools may also be suitable. We also recommend using a standardized score sheet to facilitate data analysis. An example of this has been developed and proven reliable to assist clinicians in calculating the RBT performance (13), especially accuracy score (Supplementary Material 3). This method demonstrates moderate to good reliability.

#### 2.3.3 Side hop test (SHT) + visual occlusion

Patients perform the SHT (19) as quickly as possible while wearing strobe glasses to impose visual constraints (20–22). The occlusion frequency should be adjusted according to the patient's capacity. Most strobe glasses operate within a range of 1–15 Hz, with each light phase lasting 100 ms (23, 24). As in the standard version of the test, any hop in which the patient touches the line is not counted. The total time required to complete 10 out-and-back jumps is recorded (1).

#### 2.3.4 Figure-of-8 test (F8T) + backward counting

Patients perform the F8T as quickly as possible (19), while simultaneously completing a serial subtraction task, subtracting seven from a randomly selected number between 200 and 250 (e.g., **236** ≥ 229-222-215-208-201-194.), excluding numbers ending in 7 and 0 (25). Participants are instructed to perform the motor and cognitive tasks to the best of their ability, to continue hoping even if errors occur, and to avoid prioritizing one task over the other (26). In the event of an incorrect response, the examiner immediately provides the correct answer so that the patient can continue the sequence without interruption (see more example in Supplementary Material 4). The total time required to perform the F8T, as well as the number of correct responses, are recorded.

As in the classical version of the Ankle-GO™ score (1) one additional point is awarded for each test if the patient reports no feelings of instability (19).

#### 2.3.5 Implementation and interpretation

Similarly to the Ankle-GO™ score, this “β(rain)” extension was conceptualized as a return-to-sport criterion for patients suffering from LAS, and even more so in the context of CAI. This extension appears particularly relevant for athletes involved in sports that require dual-task situations (such as team or court sports), which impose cognitive demands and where vision is primarily dedicated to managing a complex and dynamic environment.

**Figure 2:**
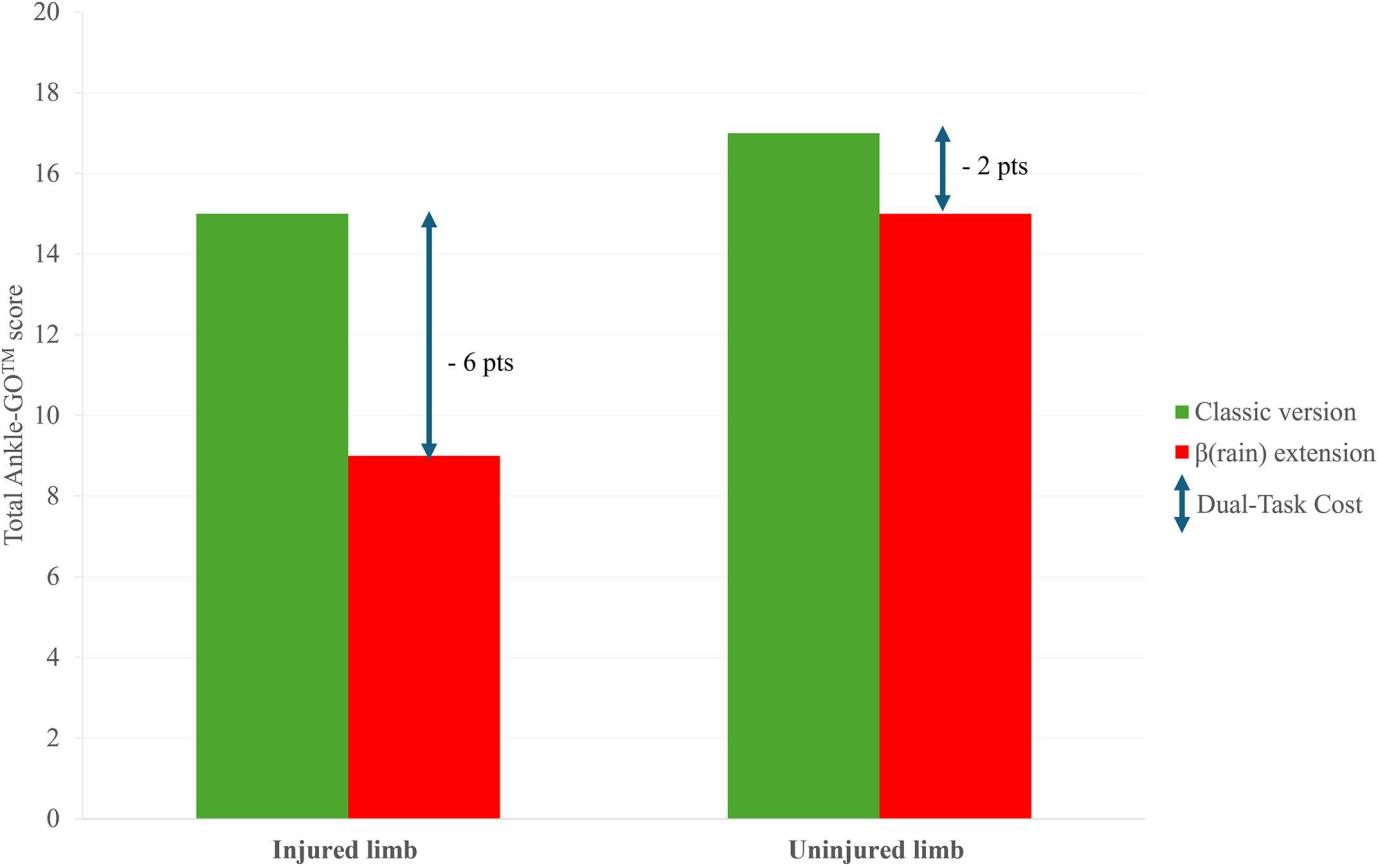
Example of possible results obtained with the classical (green) and β(rain) extension (red) of the Ankle-GO™ score for an injured and uninjured limb. The higher Dual-Task Cost (DTC) on the injured limb reflect a lower ability to manage neurocognitive constraints.

In practice, clinicians should first administer the “classical” Ankle-GO™ score, followed by the “β(rain)” extension, and then compare the results obtained between the two (Figure 2). We further recommend providing no specific instructions to patients regarding the prioritization of functional vs. cognitive tasks (16). As noted earlier, cognitive tests (e.g., digit/word span, reaction time, and backward counting) should also be performed under single-task condition (i.e., quiet sitting) during the early phase of rehabilitation to serve as a baseline and to identify prioritization (25). Since this “β(rain)” extension is performed after the classical Ankle-GO and single task cognitive constraints, no familiarisation trial is needed.

As mentioned in the first part of this topic (51), the impact of motor-cognitive interference can be quantified using Dual-Task Cost (DTC) (27).

$$\mathrm{DTC}=\frac{\mathrm{Dual}\;\mathrm{task}\;\mathrm{performance}\;\text{-}\mathrm{Single}\;\mathrm{task}\;\mathrm{performance}\;}{\mathrm{Single}\;\mathrm{task}\;\mathrm{performance}}\;\times 100$$

Limb asymmetries as well as comparisons between CAI/LAS patients and control should be reported by clinicians.

Separate DTC values for motor and cognitive domains help identify which system is compromised and how task prioritization influences performance. Differences between the 2 versions reflect the influence of cognitive and visual constraints (7, 8). Based on emerging evidence that CAI patients exhibit both cognitive and sensory reweighting deficits (51) higher DTC scores would be expected on the injured limb compared to uninjured limb or to healthy individuals. Because each test targets specific impairments (functional and visuo-cognitive), we recommend to carefully analyse all items of the score to better characterize individual deficits and optimize rehabilitation. It should be noted that specific DTC values cannot be calculated for the RBT, as no reach distance is measured, in contrast to the YBT or mSEBT. However, LED distances are directly calculated from the mSEBT (17) so it appears relevant to compare values from RBT and mSEBT in order to identify the impact of cognitive task on dynamic postural control.

In terms of rehabilitation progression, we recommend performing the “β(rain)” extension only when results on the “classical” version are considered normal by the clinician (e.g., comparable to the uninjured limb or the preinjury data). We further recommend completing the two self-reported questionnaires (FAAM and ALR-RSI) after functional tests. This sequencing provides a more accurate assessment of the patient's psychological readiness to RTS (4), as it captures patients' perceptions under game-like conditions, particularly their ability to manage dual-task demands and their confidence in ankle stability during unanticipated movements.

Finally, to facilitate interpretation of this new score, clinicians should not only consider the overall result but also examine specific items showing marked deficits. Each functional test targets a distinct capacity - for example, static postural control with the SLS, dynamic balance with the RBT, and hop/plyometric performance with the SHT and F8T. Likewise, each dual-task also engages separate cognitive domains. Identifying patient-specific deficits will allow clinicians to more effectively tailor and personalize subsequent rehabilitation and injury-prevention programs.

## 3 Discussion

This two-part article aimed to provide an overview of current knowledge on cognitive and sensory reweighting alterations in LAS or CAI patients, and to propose a practical framework for clinical evaluation to guide the late phase of rehabilitation. First, we clarified and defined the concepts of cognition and sensory reweighting. We then summarized the central deficits identified in patients and emphasized the importance of evaluating these specific impairments during the RTS phase. Finally, we reviewed the current evidence on objective criteria used in RTS decision-making, including the Ankle-GO™ score (51). However, this score does not incorporate dual-task assessments and therefore cannot capture potential central alterations. More broadly, cognitive constraints and visual reliance remain insufficiently addressed in RTS evaluations, with only a limited number of studies including such elements in LAS or CAI populations (13, 20, 28). Consequently, there is a critical need for the development of functional performance tests that integrate both cognitive and visual perturbations in dual-task situations to more accurately evaluate patients throughout the RTS continuum (6, 8, 29–31).

This second part was designed as a perspective article to provide concrete examples and practical applications of assessment for clinicians. Given the limited number of validated functional tests addressing cognitive function and sensory reweighting capacity, we propose a “β(rain)” extension of the Ankle-GO™ score to support RTS decision-making. The four functional tests—Single-Leg Stance, modified Star Excursion Balance Test, Side Hop Test, and Figure-of-8 Test—are administrated under dual-task conditions that incorporate constraints targeting key cognitive domains (i.e., attention, working memory, inhibition) and visual reliance. The combination of the tests enables the simultaneous assessment of motor performance (postural errors, maximal reach distance, time to complete dynamic task) and cognitive performance (accuracy, response time, and error rate) under dual-task conditions.

In the SLS, for example, a digit/word span task is employed to evaluate short-term verbal memory. Recent findings indicate that patients with CAI may require additional brain resources to maintain balance during single-leg stance (32). Moreover, males CAI patients exhibited reduced function related to memory and attention (31). In this dual-task extension, the performance is evaluated by quantifying both postural and digit/word span errors, thereby allowing clinicians to determine whether the patient prioritizes motor, cognitive performance, or successfully manages both). Interestingly, in healthy individuals, postural performance may remain stable—or even improve—without detriment to cognitive performance (33), suggesting efficient dual-task management. However, this phenomenon is rarely observed in patients with CAI (8).

The Reactive Balance Test is proposed as a complement to the mSEBT. This test was developed to systematically incorporate components such as decision-making, visuomotor responses, and environmental perception into the mSEBT framework (13, 17, 18). Recent evidence supports the utility of the RBT in detecting central sensorimotor impairments among individuals with CAI. The test has demonstrated moderate to excellent reliability and effectively discriminates between CAI patients and healthy controls. Notably, participants with CAI exhibited significantly reduced task accuracy without concomitant slowing visuomotor response times, suggesting deficits in processing speed and response selection when balance demands are coupled with perceptual decision-making. These findings align with the theoretical rationale of the “β(rain)” framework, which seeks to embed cognitive demands within functional testing. Accordingly, the proposed scoring system incorporates penalties for delayed response times (>800 ms), reduced accuracy (<85%) and diminished dynamic postural control. This reflects performance characteristics commonly observed in the CAI population and may assist clinicians in more accurately evaluating RTS readiness under ecological, cognitively loaded conditions.

The use of visual constraints (e.g., strobe glasses) during the SHT enables assessment of reliance on visual information during dynamic tasks (34). Stroboscopic glasses are expected to impair motor performance (i.e., reduced jump performance and increase execution time). Patients with CAI exhibit altered movement patterns and poorer dynamic postural control, including increased ankle-inversion angle and heightened peroneus longus activation during the stance phase of a landing-cutting task (23, 35). As a result, CAI patients might report feeling of instability or show significant performance declines compared to the “classical” version.

Lastly, the F8T, when combined with subtraction tasks, simultaneously targets plyometric performance as well as attention and working memory. Backwards counting stresses the phonological loop of working memory (16). Evidence shows that CAI patients exhibit poorer cognitive performance in subtraction tasks during walking compared to healthy individuals (26). Moreover, only CAI patients have been found to alter stride variability while walking-subtraction dual-task (25). CAI patients with higher self-reported disability also demonstrate poorer neurocognitive hop performance (36). Similarly, dual-task drop-landing paradigms (mental subtraction) have been shown to elicit excessive ankle inversion exclusively among patient with CAI (37). These findings highlight the clinical relevance of integrating cognitive load into functional testing, as such deficits are particularly pertinent in sport-specific contexts requiring rapid decision-making, attentional switching, and goal-directed movement under cognitively demanding conditions.

### 3.1 Implications for clinicians

This framework integrates dual-task paradigms encompassing key domains of cognitive function into established functional tests. This approach is consistent with accumulating evidence that athletic performance in real-world environments depends not only on physical readiness but also on efficient cognitive-motor control in complex and unpredictable contexts (37–39). The proposed “β(rain)” extension aims to approximate the cognitive demands of sport-specific scenarios.

In LAS patients presenting with elevated dual-task cost (DTC), emerging evidence supports the implementation of multitask paradigms (40), unanticipated movement conditions (41), and visual perturbations (42) during the late phase of rehabilitation. Multitask training enhances individuals' ability to cope with the limited processing capacity of the central nervous system (43), with particular benefits observed in CAI patients (40, 44). While conventional balance training protocols appear inefficient to reduce visual reliance during single-leg stance in CAI patients (45), the use of stroboscopic glasses during rehabilitation show promise for improving impaired sensory-reweighting strategy (i.e., increased visual reliance) (20, 21, 42, 46).

### 3.2 Futures directions

Virtual, augmented, and mixed reality (VR/AR/MR) technologies offer additional opportunities to enhance the external validity of functional assessments in individuals with LAS and CAI. These immersive technologies enable the integration of visual, cognitive, and motor challenges within controlled yet dynamic environment that replicate real-world (sport) scenarios requiring simultaneous postural control and decision-making. Evidence also suggests that VR-based training can improve static balance and perceived ankle stability; however, outcomes for dynamic balance and strength remain inconsistent compared to conventional physiotherapy. Importantly, VR environments stimulate central neural adaptations by engaging cortical regions responsible for visuomotor coordination, proprioception, and attentional control, domains frequently impaired in CAI.

Incorporating VR/AR/MR may therefore provide more challenging and realistic RTS assessments by embedding decision-making challenges, unpredictable cues, or visual perturbations into existing functional evaluations. These immersive tools also offer the potential to assess sensorimotor integration and task prioritization strategies under dual- or multi-task conditions with greater external validity than conventional testing. However, current research is limited by heterogeneity in testing protocols and outcome measures. Concerns also remain regarding the movement quality when executing functional tasks in virtual environments. Future studies should prioritize the standardization of testing procedures and explore the predictive value of VR/AR/MR-based assessments for both rehabilitation and injury prevention for LAS and CAI populations.

Integrating objective biomechanical tools such as force plates and wearable inertial measurement units (IMUs) may provide a more accurate analysis of the biomechanical effects of dual-task situations in CAI patients. Alteration in center of pressure displacement, vertical ground reaction force, peak accelerations and angular velocities of the center of mass could enable practitioners to identify cognitive or sensory-reweighting impairments with greater accuracy (47).

Dual-task measures, such as DTC, provide a valuable lens through which to examine how athletes recovering from LAS manage simultaneous motor and cognitive demands. These metrics can help clinicians detect subtle deficits that may not appear in single-task assessments and can inform the design of rehabilitation strategies that integrate cognitive load into physical training. Yet, elevated DTC values should not be interpreted as inherently pathological, as similar findings are frequently observed in healthy athletes. This underscores the limitation of DTC as a reliable predictor of reinjury risk or as an independent criterion for RTS decisions.

At present, the most appropriate role of DTC lies in shaping individualized rehabilitation content; for instance, identifying athletes who may benefit from dual-task or context-specific training. RTS decisions, however, should remain a multifactorial process, integrating objective performance measures with clinical reasoning and the athlete's unique sporting context, including competition level and sport-specific demands. Future work should continue to explore how DTC, in combination with other indicators, might contribute to more comprehensive and evidence-based RTS frameworks.

### 3.3 Study limitations

The present description of the “β(rain)” extension of the Ankle-GO™ score is proposed as a perspective and has not yet been scientifically validated. Its aim is to provide clinicians with a potential means of objectively assessing patients' performance in dual-task conditions involving cognitive load and visual disturbances. Futures studies are required using the COSMIN framework (50) in patients with CAI to assess its validity and reliability.

Three groups of participants will be recruited:

1. Healthy controls – individuals with no history of ankle sprain.
2. Chronic ankle instability (CAI) group – Patients were included only if they met the International Ankle Consortium recommended criteria for CAI (48). More specifically, patients were required to be more than 12 months from the index ankle sprain and have suffered from at least 2 recurrent sprains; report feelings of instability (Cumberland Ankle Instability Tool <24); and report loss of self-reported function (FAAMadl <90% FAAMsport <80%).
3. Copers, operationally defined as LAS patients that experienced no episodes of giving way or recurrent LAS, had a CAIT score ≥24 and returned to their preinjury sports (49).

Each group will include at least 30 participants, matched for age, sex, and level of sports activity and will perform the “β(rain)” extension.

#### Study psychometric analyses

Test–retest reliability will be assessed in 10 participants per group (CAI, Copers, Controls) who will complete the test twice, one week apart, under identical testing conditions. Intraclass correlation coefficient, Standard Error of Measurement and the Minimal Detectable Change will be calculated.

Floor or ceiling effects will be considered present if more than 15% of the participants obtain the lowest (0 points) or highest (25 points) possible total score, respectively. Discriminant validity will be assessed by comparing performances among the three groups (CAI, Copers, Controls) to determine the score's ability to distinguish between different levels of functional stability and recovery. Last, 2-month and 4-months performances will be compared among CAI patients to evaluate responsiveness of the score.

The internal consistency of the seven components of the score will be evaluated using Cronbach's alpha coefficient. Construct validity will be assessed using Pearson's correlation coefficient (*r*) to determine the strength of association between individual components and the total score.

Clinicians may consider adapting the assessment protocol to include more sport-specific tasks tailored to athlete profile (sex, type and level of play). Furthermore, normative data from healthy individuals should be established to facilitate interpretation and guide RTS decisions. Lastly, the “β(rain)” extension requires stroboscopic glasses and a reaction training system. While costs may still limit their use, the wider availability of such technologies has reduced prices and eased their integration into clinical practice (from 300 to 3,000 euros). Nonetheless, dedicated equipment remains essential for reliably assessing central alterations.

## 4 Conclusion

This perspective article proposes a practical framework for assessing patients with LAS or CAI during the late phase of rehabilitation and across the RTS continuum. The β(rain) extension of the Ankle-GO™ score was designed to address the cognitive and sensory deficits commonly observed in these populations by incorporating dual-tasks situations. Compared with classical testing, this extension offers a more ecologically valid evaluation of the central abilities required in sport-specific contexts. Clinicians are encouraged to compare functional and cognitive performance under single – and dual-task conditions, while also evaluating task prioritization in order to inform goal-oriented rehabilitation. Further studies are needed to evaluate the validity and reliability of this “β(rain)” extension in patients suffering from LAS and CAI, and to determine its capacity to identify athletes who are truly ready to return to sport.

## Data Availability

The original contributions presented in the study are included in the article/Supplementary Material, further inquiries can be directed to the corresponding author.
