# Supplementary material for: Targeting visual-sensory and cognitive impairments following lateral ankle sprains: a practical framework for functional assessment across the return-to-sport continuum. Part 2: from theory to practice: recommendations for optimizing return to sport after lateral ankle sprains using cognitive and visual-sensory assessments
Source: Front Sports Act Living. 2025 Nov 17;7:1702858. doi: 10.3389/fspor.2025.1702858 (PMC12665734; doi:10.3389/fspor.2025.1702858)
Supplement: Supplementary file 1 [file Supplementaryfile1.docx]

Supplementary Material

**
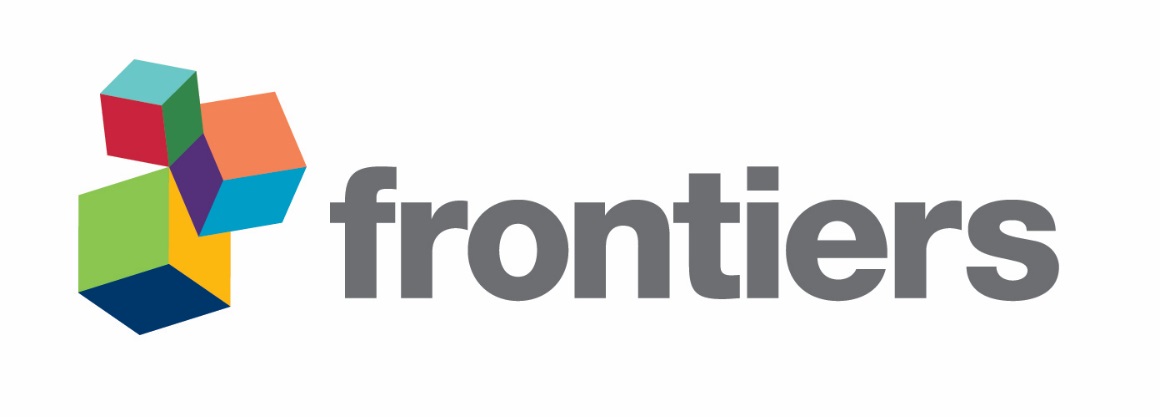
**

**Supplementary Material 1.** Example of sequence of random numbers for the dual-task (Single Leg Stance Test + digit span)

| **Instructions** | **Correct answers** |
| --- | --- |
| 1, 7, 10, 0, 2, 3 | 3, 2, 0, 10, 7, 1 |
| 2, 8, 6, 3, 4, 5 | 5, 4, 3, 6, 8, 2 |
| 6, 3, 0, 10, 8, 4 | 4, 8, 10, 0, 3, 6 |
| 7, 9, 4, 1, 10, 3 | 3, 10, 1, 4, 9, 7 |
| 3, 10, 7, 1, 6, 9 | 9, 6, 1, 7, 10, 3 |
| 6, 4, 0, 2, 9, 5 | 5, 9, 2, 0, 4, 6 |
| 2, 6, 7, 3, 1, 4 | 4, 1, 3, 7, 6, 2 |
| 3, 5, 10, 2, 0, 4 | 4, 0, 2, 10, 5, 3 |
| 0, 8, 1, 6, 9, 3 | 3, 9, 6, 1, 8, 0 |
| 2, 3, 8, 7, 4, 1 | 1, 4, 7, 8, 3, 2 |

**Supplementary Material 2.** Example of sequence of random numbers for the dual task (Single Leg Stance Test + word span)

| **Instructions** | **Correct answers** |
| --- | --- |
| Igloo-Gladiator-Venus-Compet-Larva-Prince | Prince-Larva-Compet-Venus-Gladiator-igloo |
| Yellow-Zebra-Plane-Butter-Castle-Diamond | Diamond-Castle-Butter-Plane-Zebra-Yellow |
| Train-Rainbow-Star-Tiger-Volcano-Whale | Whale-Volcano-Tiger-Star-Rainbow-Train |
| Snow-Candle-Dolphin-Ball-House-Fan | Fan-House-Ball-Dolphin-Candle-Snow |
| Koala-Lighthouse-Moon-Nest-Orchid-Helmet | Helmet-Orchid-Nest-Moon-Lighthouse-Koala |
| Apple-Dog-Notebook-Queen-Kite-Purple | Purple-Kite-Queen-Notebook-Dog-Apple |
| Dark-Ocean-Elephant-Computer-Court-River | River-Court-Computer-Elephant-Ocean-Dark |
| Swan-Oasis-Chair-Tarantula-Hole-Green | Green-Hole-Tarantula-Chair-Oasis-Swan |
| Pill-Rope-Tear-Deputy-Network-Cereals | Cereals-Network-Deputy-Tear-Rope-Pill |
| Keyboard-Table-Rabbit-Ski Boot-Blue-Hat | Hat-Blue-Ski Boot-Rabbit-Table-Keyboard |

**Supplementary Material 3.** Reactive balance test score sheet (from Maricot et al. 2024). The first column indicates the stimulus number (n=36). The target color is indicated by the color on the score sheet and stated in column 2. The following columns indicate the possible results on a stimulus: successful, missed, multiple attempts, decision error and balance error (in order).


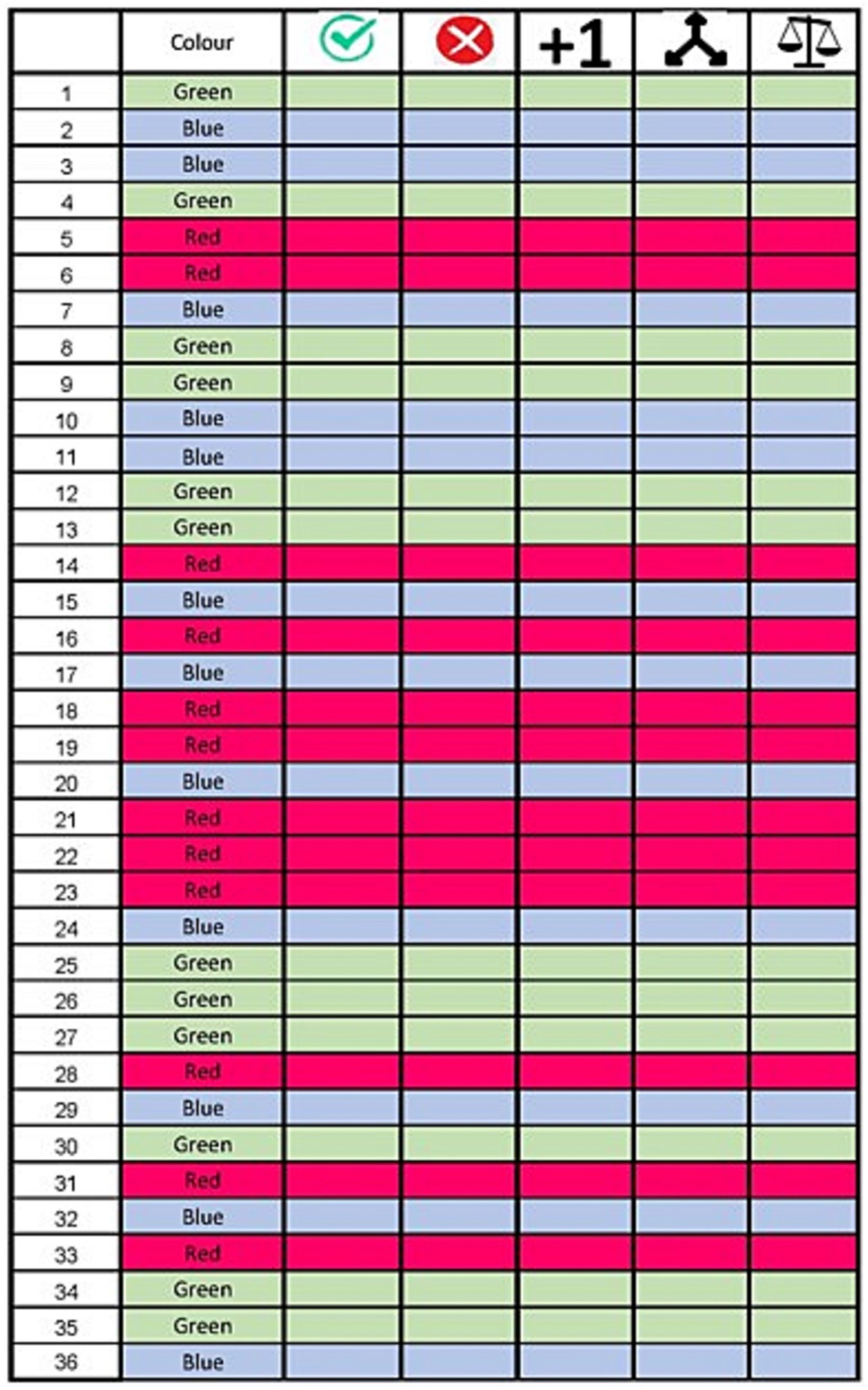


**Supplementary Material 4.** Example of sequences subtracting by 7 starting from a random number between 200 and 250 (excluding numbers ending in 0 or 7) for the Figure-of-8 test

| **Instructions** | **Correct answers** |
| --- | --- |
| **236** | 229, 222, 215, 208, 201, 194, 187, 180, 173 |
| **203** | 196, 189, 182, 175, 168, 161, 154, 147, 140 |
| **225** | 218, 211, 204, 197, 190, 183, 176, 169, 162 |
| **248** | 241, 234, 227, 220, 213, 206, 199, 192, 185 |
| **213** | 206, 199, 192, 185, 178, 171, 164, 157, 150 |
| **222** | 215, 208, 201, 194, 187, 180, 173, 166, 159 |
| **209** | 202, 195, 188, 181, 174, 167, 160, 153, 146 |
| **235** | 228, 221, 214, 207, 200, 193, 186, 179, 172 |
| **211** | 204, 197, 190, 183, 176, 169, 162, 155, 148 |
| **245** | 238, 231, 224, 217, 210, 203, 196, 189, 182 |
